# Sulphuric Ether in Surgical Operations

**Published:** 1847-06

**Authors:** James Bryan

**Affiliations:** Lecturer on Surgery; formerly Professor of Surgery and Medical Jurisprudence, in Castleton Academy of Medicine, Vermont


### MEDICAL EXAMINER,

AND

### RECORD OF MEDICAL SCIENCE.

NEW SERIES.—No. XXX.—JUNE, 1847.

---

### ORIGINAL COMMUNICATIONS.

*Sulphuric Ether in Surgical Operations.* By JAMES BRYAN, M. D., Lecturer on Surgery; formerly Professor of Surgery and Medical Jurisprudence, in Castleton Academy of Medicine, Vermont.

To the Editor of the Medical Examiner.

Mrs. A——, a married lady, of delicate health and constitution, who had suffered for a long time from neuralgia and facial inflammations, caused by decayed teeth, consented to try the ether as administered by Mr. H. S. Porter, dentist, and one of my pupils. The inhalation was made from the instrument called the "*Inhalus*" of the patentees. About half an ounce of the ether was poured into the sponge, and the lady, seated in an arm chair, inhaled the vapour about five minutes, when she became languid and sleepy, and desired to be 'let alone.' The instruments were applied, and three teeth (two incisors and one cuspid) were dexterously extracted; the patient only raising her hand slowly, and drawing a deep sigh on the extraction of each tooth. The immediate effects of the ether continued about three or four minutes, when she expressed herself surprised that the teeth had been taken out.

Miss B——, on the 2d of April, inhaled the vapour from the same apparatus in my parlour, and after continuing the process about eight minutes, appeared partially insensible, and had a dens sapientiæ of the upper jaw extracted by the same operation, and said that she experienced no pain.

A few days after this, Mrs. A., the first patient, took the ether again, inhaling it about five minutes before the soporific effects were produced, when Mr. P. extracted two of the largest molars of the upper jaw, and the remaining *cuspid* of the same jaw. The *lethean* effect had passed off before the last tooth was extracted, and she said that she experienced considerable pain during the extraction, but much less than if no ether had been administered. A slight head ache, which may in part be attributed to previous illness, continued for four or five hours.

On the 7th of April, Miss C——, from Massachusetts, in the presence of Dr. Winslow, myself, and members of the family in which she resided, submitted to the operation for extirpation of the left mamma, while under the influence of the ether. The inhalation, under the direction of Mr. Porter, continued about eight minutes, when I made two elliptical incisions, including the nipple, and took out the tumour in the usual manner. The disease being cancerous, a portion of the healthy part was included. I proceeded, with the assistance of Dr. Winslow, to take up the arteries, when the patient inquired if there was any more cutting to be performed, seemingly unconscious that the tumour was removed. She declared, however, that she was conscious of each incision that was made, but felt *no power* to *resist*. The larger vessels were taken up, and the wound left partly dressed, that time might be allowed for reaction fairly to take place. Rather more than half an hour afterwards reaction did take place, and the vessels not taken up bled profusely, making it necessary to remove the dressings and carefully take up the most minute arteries. After this, the wound was properly dressed, and did very well, healing up almost entirely by the first intention.

Miss C. says, since she has reflected upon it, that she is sure the greatest part of the pain was avoided—very little, in fact, having been suffered. She was desirous of taking the ether while the arteries were being taken up, but I would not consent, being convinced that one effect of the ether was to diminish the action of the arterial system.

I send you these cases, sir, without comment. These—with one other case in which the patient insisted that the tooth had not been taken out, although the operation was slow and difficult--are all I have seen, and from these I am *rather* disposed to try *it again in certain cases*.

